# The γ^33^ subunit of R-phycoerythrin from *Gracilaria chilensis* has a typical double linked phycourobilin similar to γ subunit

**DOI:** 10.1371/journal.pone.0195656

**Published:** 2018-04-10

**Authors:** Aleikar Vásquez-Suárez, Francisco Lobos-González, Andrew Cronshaw, José Sepúlveda-Ugarte, Maximiliano Figueroa, Jorge Dagnino-Leone, Marta Bunster, José Martínez-Oyanedel

**Affiliations:** 1 Laboratorio de Biofísica Molecular, Departamento de Bioquímica y Biología Molecular, Facultad de Ciencias Biológicas Universidad de Concepción, Concepción, Chile; 2 Michael Swann Building, Kings’ Buildings, University of Edinburgh, Edinburgh, Scotland, United Kingdom; Nagoya University, JAPAN

## Abstract

Phycobilisomes (PBS) are accessory light harvesting protein complexes formed mainly by phycobiliproteins (PBPs). The PBPs absorb light that is efficiently transferred to Photosystems due to chromophores covalently bound to specific cysteine residues. Besides phycobiliproteins (PE), the PBS contains linker proteins responsible for assembly and stabilization of the whole complex and the tuning of energy transfer steps between chromophores. The linker (γ^33^) from *Gracilaria chilensis*, is a chromophorylated rod linker associated to (αβ)_6_ hexamers of R-phycoerythrin (R-PE). Its role in the energy transfer process is not clear yet. Structural studies as well as the composition and location of the chromophores are essential to understand their involvement in the energy transfer process in PBS. To achieve this, the coding gene of γ^33^ was cloned and sequenced. The sequence was analyzed by informatics tools, to obtain preliminary information which leaded the next experiments. The protein was purified from R-phycoerythrin, and the sequence confirmed by mass spectrometry. The coding sequence analysis revealed a protein of 318 aminoacid residues containing a chloroplastidial transit peptide (cTP) of 39 aminoacids at the N-terminus. The conservation of cysteines revealed possible chromophorylation sites. Using α and β R-PE subunits as spectroscopic probes in denaturation assays, we deduced a double bonded phycourobilin (PUB) on γ^33^ subunit that were confirmed between Cys62 and Cys73 (DL-PUB^62/73^) by mass spectrometry. The cysteines involved in the double link are located in a helical region, in a conformation that reminds the position of the DL-PUB^50/61^ in the β subunit of R-PE. The position of single linked PUB at Cys^95^ and a single linked PEB at Cys^172^ were also confirmed. Spectroscopic studies show the presence of both types of chromophores and that there are not energy transfer by FRET among them.

## Introduction

Phycobilisomes (PBS) are highly efficient accessory light harvesting protein megacomplexes, responsible for the conduction of energy towards the photosynthetic reaction centers present in cyanobacteria and red algae. They absorb light in a wavelength range where chlorophyll is poorly efficient (the green gap), and transfer energy directionally to the photosystem II [1]. This is possible thanks to phycobiliproteins (PBPs) and chromophores (lineal tetrapyrrols) covalently attached at specific cysteine residues. All PBPs share a general architecture: they are formed by a basic αβ heterodimer, which has been shown to oligomerize to (αβ)_3_ trimers (allophycocyanin) or (αβ)_6_ hexamers (phycocyanin and phycoerythrin), acquiring a ring structure. *Gracilaria chilensis (G*.*ch*) phycobilisomes contain allophycocyanin (APC, λ^A^ max 651nm) in the core of complex, from where radiate rods, formed by phycocyanin (PC, λ^A^ max 621nm), closer to core and phycoerythrin (PE, λ^A^ max 565 nm) at the distal end of the rods [2, 3]. They have their absorption and emission spectra overlapped, allowing a non-radiative, direct and efficient transfer of the excitation energy among them, which is channeled along an energy gradient from the rods to the core and finally transferred to chlorophyll *a* in photosystem II [4].

Efficient energy transfer is achieved through a combination of the position, geometry and spectroscopic properties of chromophores, and the protein environment in which they are placed. Besides PBPs, they include linker proteins responsible of the assembly and stabilization of the whole complex, and the fine-tuning of the energy transfer steps between chromophores [5]. The linker proteins are located within the rods (L_R_), rod-core interface (L_RC_), core (L_C_) and core-membrane interface (L_CM_). Although most linker proteins are colorless, chromophore bearing linkers have been described, which suggest their direct participation in the energy transfer process. The 31 and 33 kDa γ subunits of R-PE (γ^31^) and (γ^33^) respectively, are two examples of them. They form complexes with (αβ)_6_ hexamers of R-PE [6, 7]. Regarding their spectroscopic properties, both linkers have two absorption maxima, with λ^A^ max at 495nm and 555nm. Both protein sequences carry phycourobilin (PUB) and phycoerythrobilin (PEB) chromophores, just like the β subunit of R-PE, but in different proportions [6]. Each reported γ subunit, bear at least four chromophores, which could facilitate the energy transfer within the PBS rods [6], similarly to PE β subunit. The β subunit of R-PE contains two PEB bound to Cys^82^ and Cys^158^ (β-PEB^82^, β-PEB^158^) and one phycourobilin (PUB) doubly linked to Cys^50^ and Cys^61^ (β-DL-PUB^50/61^) [8].

Most phycobilisome genes are encoded in the chloroplast genome [9]. However, the genes of the γ subunits are located in the nuclear genome. Thus, in order to be imported to the chloroplast, γ subunits should bear chloroplast transit signal peptides (cTP), which the mature protein lacks [6]. Genes sequences available from different species show that the γ subunits have no introns [10], and that they do not show significant sequences identity with any of the PBP subunits nor with other linker proteins. Depending on the phycoerythrin kind, different γ subunit families have been described and, in cyanobacteria, γ subunits from *Synechocystis* and *Prochlorococcus* strain, have been characterized. In red algae, different γ subunits have been found according to the PE hexamer position in PBS rod [11, 12]. It has been proposed that linkers are positioned in the central cavity of PBP trimers or hexamers [13]. This location would disrupt the symmetry of the possible energy transfer pathways between chromophores of the complexes, guiding and promoting the energy transfer along the rods and core [14]. However, due to the symmetry displayed by the crystallographic structure of (αβ)_6_ hexamers of B and R-PE, the electronic densities associated to the γ subunits located in the inner cavity are averages and only small peptides belonging to the linker, nearby at Cys82 chromophore on β-subunit of R-PE, could be resolved [8, 15,16].

No structural information was available regarding γ subunits associated to PE, until the work recently published by Zhang *et al*. (2017) [12], from cryo-electron microscopy data, for the PBS of *Griffithsia pacífica*. In that work, the position and general structure of γ subunits in the center of the PE hexameric ring was confirmed. For our studies on the energy transfer process in PBS of *G*.*ch*, the information on the position, conformation and identity of the chromophores is also necessary and it is the main purpose of this work. The lack ofspecific atomic information about γ subunits associated to PE, makes difficult to incorporate these linkers into our energy transfer model in PBS from the red alga *G*.*ch* [17]. Therefore, although we could identify both γ subunits, in this work we present the sequence and spectroscopic information on γ^33^. This report presents also the position in the sequence and identity of the chromophores, information that, with the structural data provided by Zhang *et al*, (2017) [12] would allow us to advance in the elucidation of functional aspects of γ subunits on our energy transfer model in PBS.

## Materials and methods

### Gene amplification and sequencing

Total DNA from *G*.*ch* was collected following Hu *et al*., (2004)[18] recommendations with slight modifications. Briefly, 100 mg of fresh algal tissue was frozen with liquid N_2_ and crushed into a fine powder. After a lysis procedure with 600μL of Nuclear Lysis Buffer (Promega, EEUU) and incubation at 65°C for 15min, 3μL ribonuclease A (10 mg/mL) was added and the mixture was incubated at 37°C for 45min. The proteins in solution were precipitated by adding 200μL of 9M ammonium acetate under 15s of strong shaking. After centrifugation (3min x 13000g), the supernatant containing DNA was carefully removed. The DNA precipitation was performed by addition 600μL isopropanol at room temperature and mixed gently by inversion until DNA strands appeared. Subsequently, it was centrifuged for 1min at 13000g. The pellet was washed with 70% ethanol at room temperature by gently inverting the tube repeatedly. After centrifuging for 1min at 13000g at room temperature, the ethanol was discarded carefully and the tube inverted on absorbent paper to be dried for 15min. Finally, the pellet was suspended in 50μL of nuclease-free water. The γ^33^ subunit gene was amplified by PCR using specific forward γ^33^ (5-ATGGAAGCCCCCGCTTTTGCTG-3) and reverse γ^33^ (5-TCAGTCGCGGCACATGGCGG). The primers were designed analyzing genetic sequences and EST from red algae available at the web site of The National Center for Biotechnology Information (https://www.ncbi.nlm.nih.gov). The C-terminal region was obtained by 3´ end cDNA Amplification [19] using the Invitrogen RACE-PCR kit. The PCR products were extracted from 1% agarose gel, and the sequence of the amplified genes was determined by automatic sequencing at IDT (Integrated DNA Technologies).

### *In silico* sequence characterization

The nucleotide sequences of the γ subunits genes were translated *in silico* by ExPASy Translate (http://expasy.org). Putative chromophorylation sites were found through a multiple sequence alignment using T-Coffee [20] of γ^33^ subunits from red algae and experimentally determined chromophorylation sites from *Gastroclonium coulteri* [21] and *Griffithsia pacifica* [12]. Secondary structure features were calculated with PSIPRED [22]. Subcellular localization and the presence of chloroplast transit peptides were checked with TargetP [23] and ChloroP [24], respectively. Internal sequence repeats were identified with the *de novo* prediction server HHrepID [25].

### Purification and characterization of γ^33^ subunit

**Fig 1:**
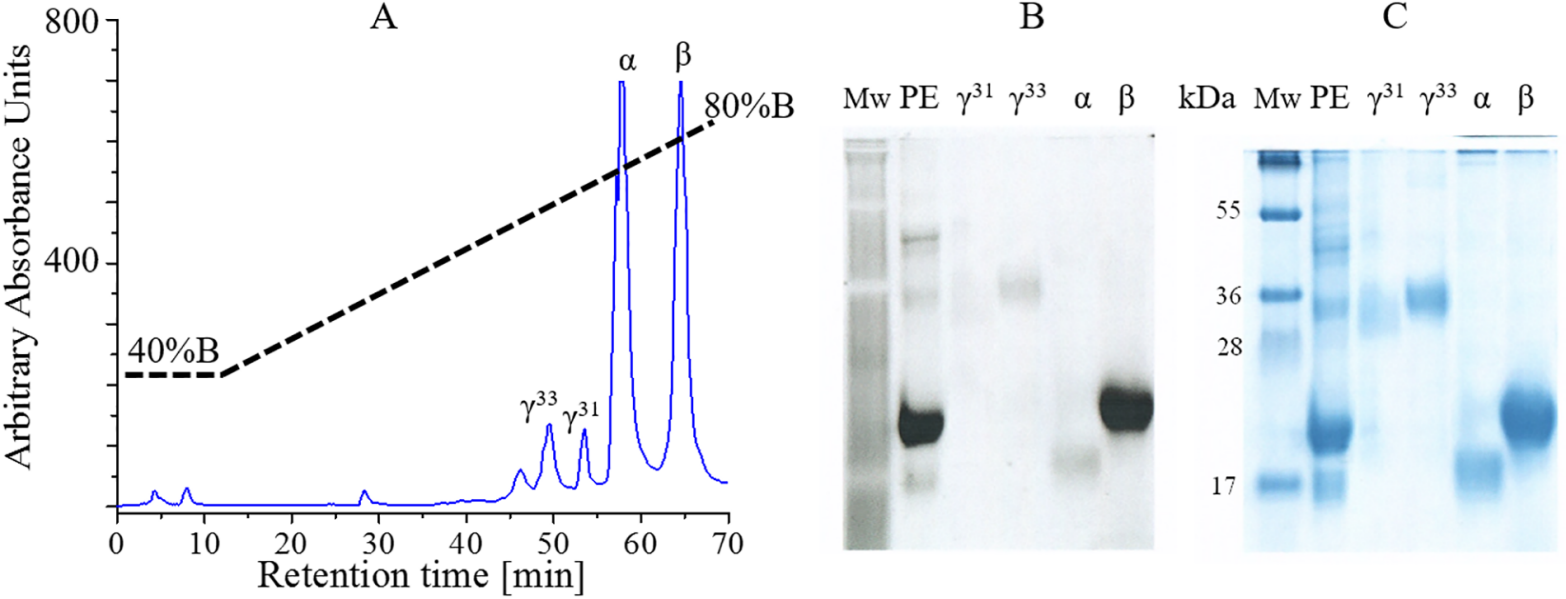
Purification of γ^33^. (A) Chromatographic separation of the subunits components of R-phycoerythrin complexes from *G*. *ch*. by RP-HPLC (B) Electrophoretic assay of the purified subunits, in 10% SDS-Tricine Gel, stained with Coomassie blue (right) and exposed to UV light to determine presence of chromophores (left).

R-PE from *G*. *ch* was purified as described previously [2] and the separation of subunits was performed as reported by Apt *et al*. (1993)[6] and Sepúlveda Ugarte *et al*. (2011)[26], with some modifications as follows: 100 μl of the purified R-PE (1 mg/mL) (A_566_/A_280_ = 4.92) were used to separate γ^33^ subunit and other proteins from PE complexes by reverse phase high-performance liquid chromatography (RP-HPLC) using a Merck-Hitachi chromatographer coupled to a C18 Lichrocart 250–4 column. The elution was performed with a gradient of solution A: 0.1% TFA in water, and solution B: 0.1% TFA in acetonitrile:isopropanol = 2:1, at a flux of 0.8 ml/min. Information regarding the gradient used are shown in Fig 1A. The elution was followed at 280nm using an UV-Vis detector. All the RP-HPLC fractions were characterized by their absorbance (Jasco V-650 spectrophotometer) and emission (Shimadzu RF-5301PC spectrofluorophotometer) spectra. Furthermore, an individual band from 10% Tricine-SDS-PAGE [27] corresponding to γ subunit was excised for analysis by mass spectrometry (ESI-FTICR) at the University of Edinburgh (IGMM MS Facility).

### Denaturation assay of purified R-PE subunits

RP-HPLC fractions of γ^33^, α and β-PE subunits were characterized by their absorption spectra, immediately after purification. Then the organic solvent present in the fractions was removed by dialysis against 10mM potassium phosphate buffer, pH 8.0 and their absorption spectra were recorded again(native spectra). Each sample was concentrated and loaded into SDS-PAGE-tricine electrophoresis as above; the bands in the gel without staining, were excised. These bands were immersed in 10 mM pH7.4 potassium phosphate buffer, supplemented with 3M guanidinium chloride for elution by agitation during 24h at room temperature. The absorbance spectra of the eluted fractions samples were also recorded (denatured spectra).

### Experimental determination of the position of double linked phycourobilin

Spectroscopic analysis plus mass spectrometry of purified chromopeptides following sequential treatments of trypsin and cyanogen bromide on γ^33^, were used to determine the sequences containing modified cysteines [28, 29, 30]. The protocol was as follows: 1μL of 1M HCL was added to 200 μL of purified γ subunit (0.5 mg/mL) with 5 μL trypsin (0.4mg/mL) and 15μL of 1M (NH_4_)HCO_3_. The mixture was then incubated at 37 °C for 2h in the dark. The mixture was thoroughly dried with Speed-Vac. 50μl of cyanogen bromide and 50% acetonitrile/isopropanol/1% trifluoracetic acid was added to the dried mixture and maintained at room temperature for 14h in the dark. The chromopeptide mixture was separated by RP-HPLC with the same separation conditions used for the constituent subunits of the hexameric complex described above. The purified proteolytic fragments obtained after digestion, were characterized by spectroscopy and analyzed by Mass Spectrometry (MS) at the University of Edinburgh. MS-bridge prospector platform (http://prospector.ucsf.edu) was used for search and identification. The elemental composition of modified cysteines and/or the chromophore as crosslinking agent were used for identification of the position of chromophore double linked. In parallel, mass spectrometry of trypsin proteolytic peptides of cross linked RPE-γ^33^(di-succinimidyl suberate (DSS) or pymelic acid hydrazide(PDH)) was also performed to identify other chromophore positions in γ^33^.

## Results and discussion

### γ^33^ was purified from R-phycoerythrin

Four subunits of R-phycoeythrin (R-PE) from hexameric complex previously purified, were separated by RP-HPLC (Fig 1A). Each of the fractions, characterized spectroscopically, was resolved by SDS-PAGE corresponding to α, β, γ^31^ and γ^33^ with 18, 20, 31 and 33 KDa of relative mass respectively, which migrate as fluorescent diffuse bands (Fig 1B). Because the amount of purified γ^31^ was insufficient for further analysis, the results and discussion are focused on γ^33^ subunit. γ subunits seemed to form a very stable complex with hexameric R-PE; they are extracted together and they co-purified. The purified γ^33^ fraction was used for the identification by mass spectrometry and for spectroscopy experiments.

### Sequence analysis of γ^33^

**Fig 2:**
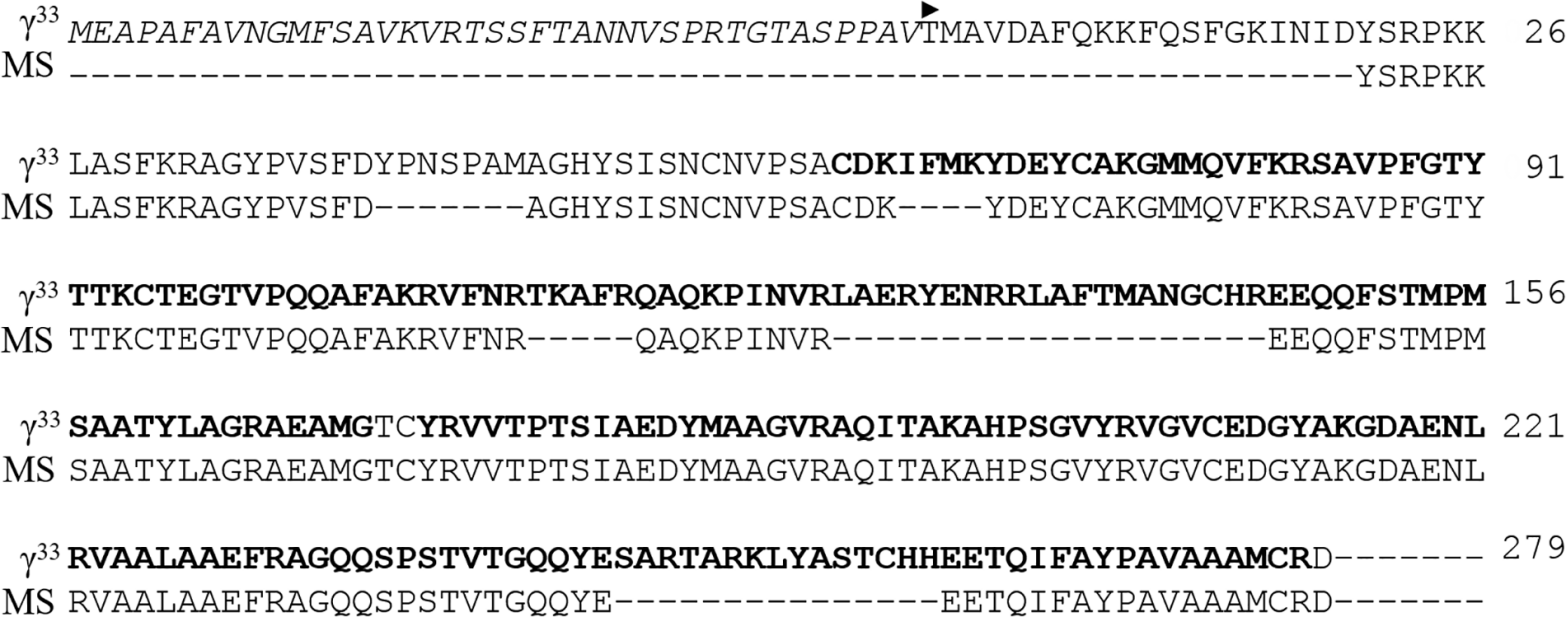
*In silico* translation of phycoerythrin γ^33^ subunit from *G*. *ch*. MS corresponds to the sequence confirmed by mass spectrometry. The chloroplast transport peptide at N-terminal is shown in cursive letters. The internal repeats are highlighted in bold letters. The arrowhead represents the start of the sequence of the mature protein.

The genomic sequence for γ^33^ was deposited under the code BankIt2062880 Seq1 MG520097 at the Gene Bank. The codifying regions were translated and compared with the sequences of γ subunits from other species. The translated amino acid sequence for γ^33^ with a chloroplast signal sequence involving the first 39 amino acids is shown at Fig 2.

The mature protein is 279 residues long (MW: 34,884 Da). The 74% of the sequence was confirmed by mass spectrometry (MS) and are displayed also in Fig 2 as MS. The sequence analysis of the protein revealed similarities between the N-terminal and the C-terminal region, showing repetitions, (Repetition I: Cys^62^-Gly^170^, and Repetition II: Tyr^173^-Arg^278^). The repetitions detected between the N-terminal and C-terminal region of γ^33^ from *G*. *ch*. are also present in γ^33^ subunit from *A*. *neglectum* [9] and in all sequences reported so far. These internal repeats often correspond to structural and functional units and could have been originated from intragenic duplication events as it was proposed for phycobiliproteins previously [11], in order to increase the number of chromophorylation sites. The occurrence of multiple phycoerythrobilins (PEBs) and phycourobilins (PUBs) [31, 32] on γ subunits is an evolutionary adaptation to habitats with low light, because the chromophore density would be increased without expanding PBS. It is also possible that the overall rate of energy transfer in peripheral rods could be increased by the presence of the bilins on the linker proteins, which only could be confirmed when the relative position and configuration of each chromophore is available.

The internal repeats in γ subunits, have been recently termed by Zhang *et al*., (2017)[12], as conserved chromophore binding domains (CBDγ) containing about 210 residues. In *G*. *pacifica*, the CBDγ consists of ten α-helices (H1-H10), which are organized in two domains: a five N-terminal helices (H1-H5) and a C-terminal five helices (H6-H10) corresponding with the two internal repetitions in γ subunits. These two domains are related by a 180° rotation around an axis parallel to the hexamer plane. They report that CBDγ would be making extensive contacts in a symmetrical fashion with the inner face of the rod hexamer [12].

### There is not energy transfer from PUB to PEB in γ^33^ subunit

**Fig 3:**
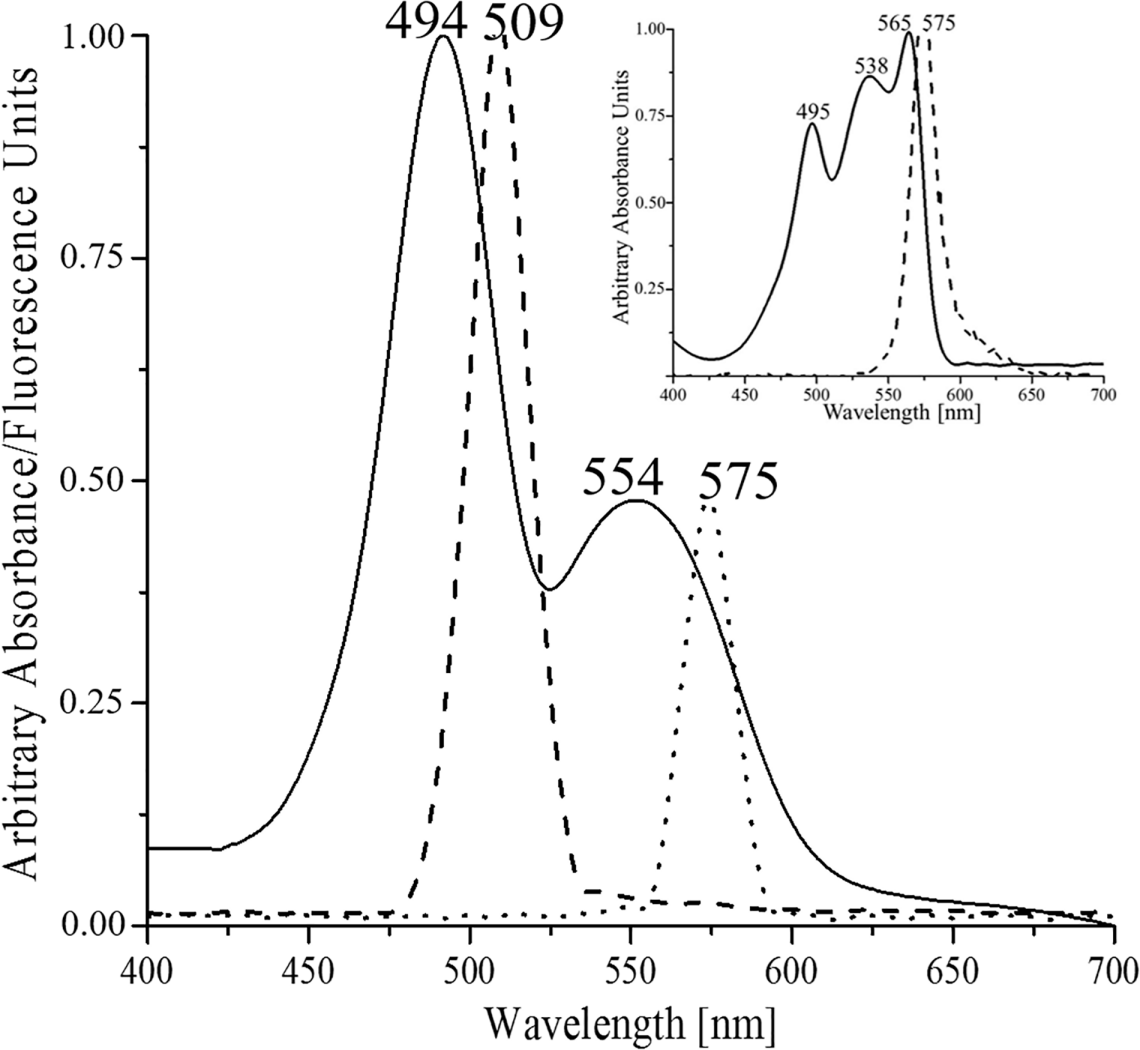
Absorption and emission spectra of the purified γ^33^. Continuous line shows the absorption spectrum. Dashed and dotted lines show the emission spectra of γ^33^ subunit after excitation at 490nm and 538nm respectively. Insert emission spectrum of R-Phycoerythrin after excitation at 490 and 540 nm.

The area ratio of the absorption peaks for PUB and PEB in the β subunit spectrum is 1:2 corresponding with (1PUB, 2PEB); for the γ^33^ subunit, the ratio was 1:1 corresponding with (2PUB, 2PEB). R-PE shows a maximum fluorescence emission at 575nm during the excitation at 490nm, indicative of energy transfer from PUB to PEB chromophores on all subunits components of the R-PE hexameric complex. However, when the purified γ^33^ was excited at 490nm or 538nm, (Fig 3), an emission fluorescence maximum for each excitation was detected, at 509nm and 575nm, respectively. This spectrum indicates that PUB present on γ^33^ should have distances or orientations that prevent the energy transfer to PEB on γ^33^, possibly favoring the energy transfer to chromophores on R-PE. This proposal can be supported first, by the crystallographic data in which γ^33^ subunit interacts with the internal region of R-PE-hexamer [8] and second, the position of R-PE chromophores Cys^82^ of α-PE and β-PE subunits, also located at inner side of PE-hexamer, [8, 15, 16].

### Chromophorylation sites on γ^33^ subunit of R-phycoerythrin of *Gracilaria chilensis*

**Fig 4:**
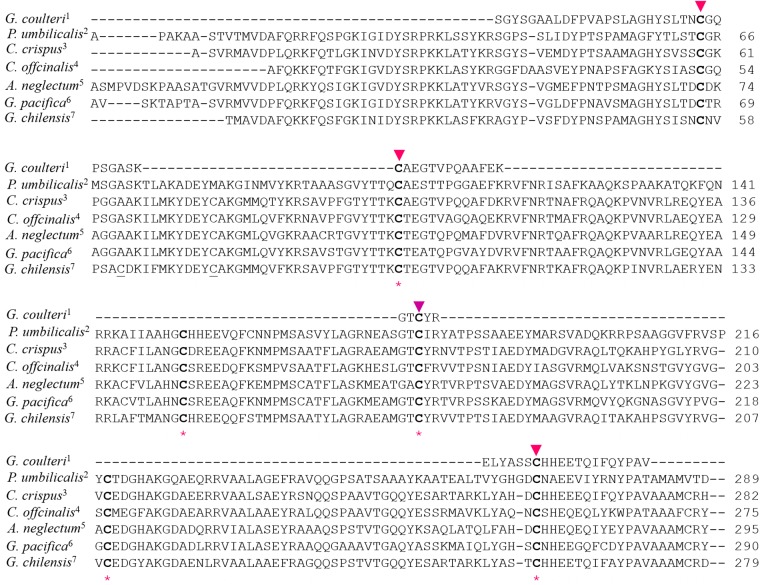
Multiple sequence alignment of γ^33^ subunits available, with the phycoerythrin γ^33^ subunits from of *G*. *ch*. In bold, the possible chromophorylation sites for conservation of cysteine residues demonstrated in *G*. *coulteri* (upper triangles) and *G*. *pacifica* (lower asterisk) are shown. 1 [21], 2 [33], 3 [34], 4 [10], 5 [6], 6 [12], 7 This work.

Zhang *et al*., (2017)[12] mentioned that the CBDγs (here internals repeats) of most γ subunits bear five bilins, with each half binding two bilins and that the remaining bilin would be located at the interface between the two halves. The exception is the CBDγ of linker rod γ7, which contains only four bilins on *Griffithsia pacifica* [12]. Nervertheless, according our alignment the first bilin (Cys^56^) is outside of internal repeat. Futhermore, the experimental work of Klotz & Glazer (1985) [21] on *G*. *coulteri*, also indicated four chromophorylated cysteines identified by mass spectrometry analysis of tryptic fragments. A multiple sequence alignment for these sequences (Fig 4) show the possible chromophorylation sites indicated by the cysteines in bold letters according to Klotz & Glazer (1985) [21] and Zhang *et al*., (2017)[12], but most of the sequences available contains a larger number of cysteines conserved. From the multiple sequence analysis (MSA) the possible chromophorylation sites for γ^33^ in *G*. *ch* should be Cys^56^, Cys^95^, Cys^172^, Cys^209^ and Cys^259^.

The MS analysis of crosslinked proteolytic fragments from γ^33^ associated to hexameric R-PE locates a single linked PUB (SL-PUB) and a single linked PEB (SL-PEB) to Cys^95^ and Cys^172^, respectively. According to the information from Klotz & Glazer (1985)[21] and Zhang *et al*. (2017)[12] and MSA with other red algae γ subunits available (Fig 3), the possible position for a SL-PEB should be Cys^259^. To conserve the ratio PUB/PEB it is necessary find the binding site for one PUB.

### There is a phycourobilin double linked (DL-PUB) on γ^33^ subunits

**Fig 5:**
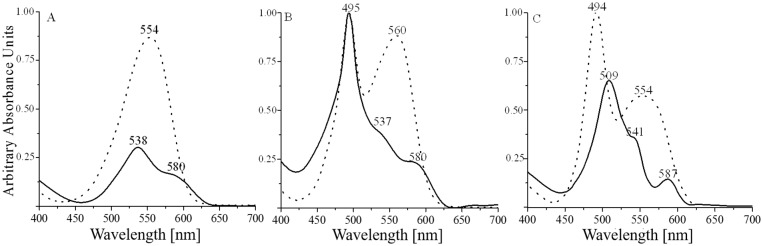
Absorption spectra of native and denatured R phycoerythrin subunits. A) α subunit, B) β subunit and C) γ^33^ subunit. Dotted line: native sample; solid line: denatured sample.

It has been reported that β-PE subunit of R-PE contains one DL-PUB bound to Cys^50^ and Cys^61^, keeping the chromophore in an extended conformation regardless the denaturation conditions and without major changes on its absorption properties [16]. The structural information on R-PE [8] confirmed a DL-PUB to cysteines in the β subunit. Previous experiments have shown also, that the DL-PUB present in R-PE is more stable to denaturation than chromophores with a single linkage [16, 35]. In order to demonstrate this last statement, the purified subunits α, β, and γ^33^ were denatured as described in methods. The absorption spectra of each subunit in the native as well as in denatured condition, are shown on Fig 5.

The α subunit of R-phycoerythrin (α-RPE), contains two single linked phycoerythrobilins (SL-PEB), bound to Cys^82^ and Cys^139^. Its absorption spectrum (Fig 5A) shows one maximum (554nm) in native state, but after the denaturation, this signal is not only reduced to a 50% intensity but two components are clearly displayed (538nm and 580nm), result that can be explained by changes in the environment in both SL-PEB. The β subunit contains two SL-PEB bound to Cys^82^ and Cys^158^, and one DL-PUB (Cys^50/61^). Its absorption spectrum in native state (Fig 5B) presents a peak at 495nm assigned to DL-PUB, and a peak at 560nm assigned to SL-PEBs. After denaturation, the 560nm maximum almost disappeared revealing two shoulders at 537nm and 580nm corresponding to SL-PEB on β subunit, similar to the change observed in α subunit. No major change was observed for the maximum at 495nm corresponding to the DL-PUB. The same denaturation assay was performed in the γ^33^ subunit. After denaturation the maximum at 494nm maintains significant absorption values with a redshift of 15nm; also a shoulder at 541nm and a decrease in the absorption at 587nm are evident. The significant permanence of the major absorbance associated to PUB, although with red shift, evidence some similarity with the behavior of the DL-PUB in β subunit of R-PE, suggesting the presence of a DL-PUB in γ^33^ subunit. The shoulder at 541nm besides the maximum at 587nm, also suggests the presence of two PEB populations like in the β subunit of R-PE (Fig 5C).

The red shift in γ-DL-PUB, (between native and denatured state or PUB-chromopeptide) may be due to interactions or direct effect of the amino acid in the vicinity of PUB which could modify the chromophore vibrational energy state. Braiman *et al*. (1999)[36] indicate that charged side chains, buried as part of an ion pair within hydrophobic regions of a protein, are more sensitive to perturbations such as changes in the nature or position of counterions, altering the formation hydrogen bonds. Bathochromic shifts up to 40nm, such as the observed in the denaturation process for PUB have been also observed by Arciero *et al*. (1988) [29] after tryptic digestion of phycobiliproteins or adducts formed *in vitro*. The DL-PUB present in β-RPE of *G*.*ch* in the native state is exposed to solvent. The DL-PUB present in γ^33^ should be more protected from the environment in the native state, this difference could explain the red shift observed upon the denaturation procedure on γ^33^.

**Fig 6:**
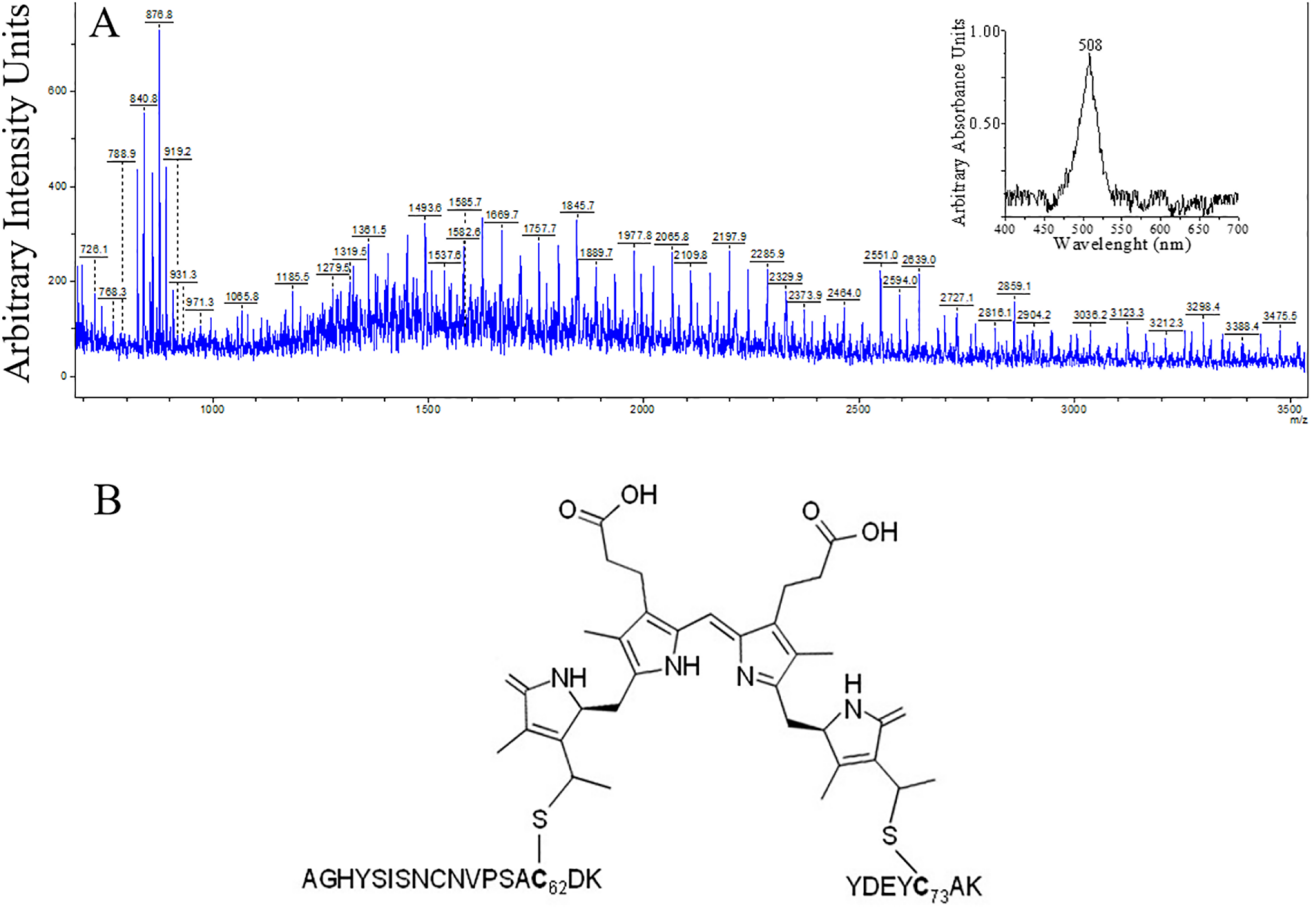
MALDI-TOF mass spectra of PUB-peptide. A) Peptide isolated from tryptic digestion and cyanogen bromide cleavage of native γ^33^ phycoerythrin. The insert shows absorption spectrum of the PUB-peptide. B) Structural scheme of PUB-peptide identified by MS-Bridge of the double linked PUB to cysteines on γ^33^.

The MS analysis (S1 Table) of the proteolytic fragment characterized as PUB-chromopeptide allowed the experimental confirmation of a DL-PUB (double linked PUB) at Cys^62/73^. The MALDI-TOF result is shown on Fig 6 in which tryptic peptides cross-linked by PUB were identified. This PUB-chromopeptide showed an absorption maximum at 508nm, the maximum for PUB, very similar to the maximum observed on denatured γ^33^ subunit (509nm).

**Fig 7:**
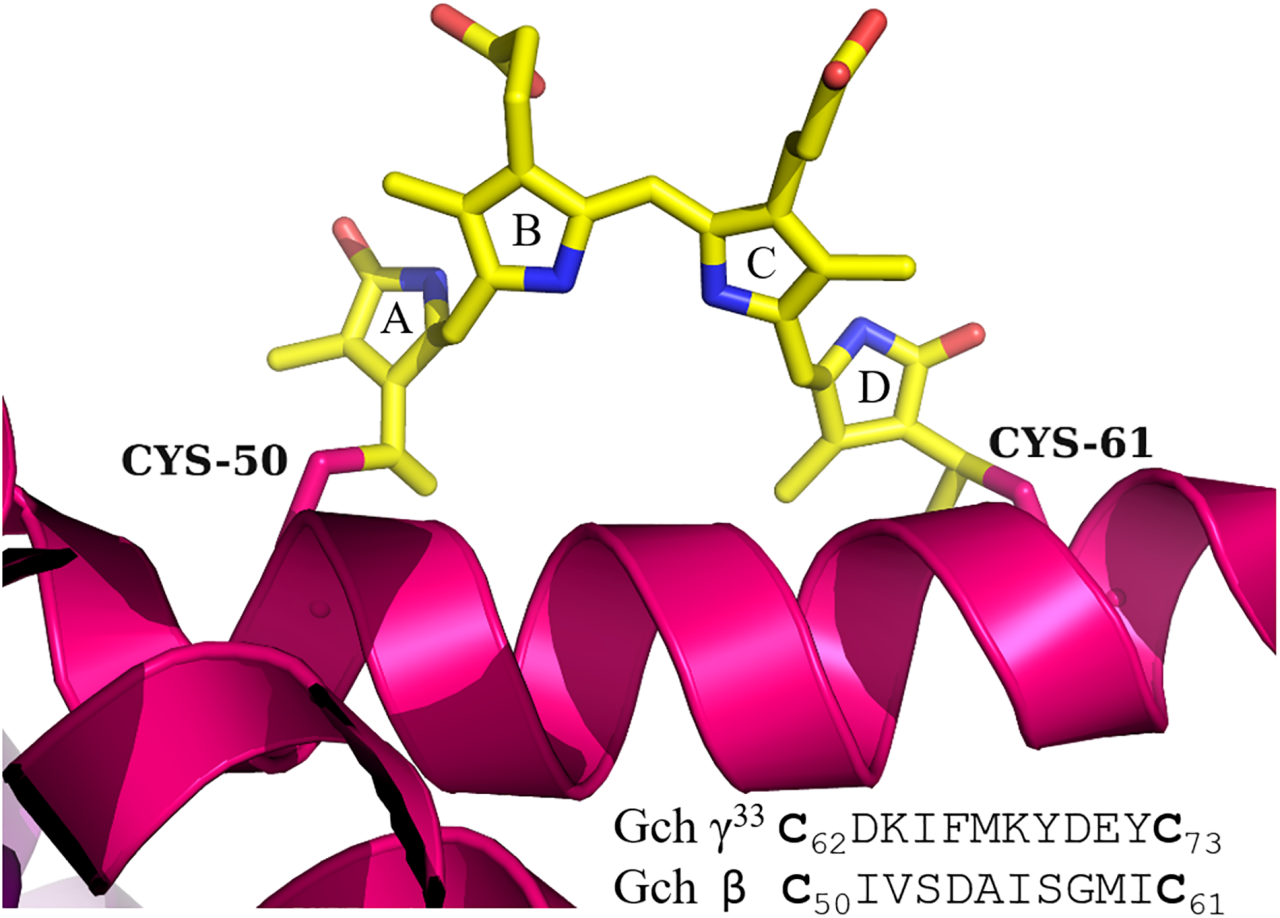
Detail of B-helix on β-PE subunit from crystallographic model PDB-1EYX. The sequences involving on β-DL-PUB^50/61^ and γ^33^-DL-PUB^62/73^ are shown for comparison.

A closer analysis of the DL-PUB in the structure of R-PE of *G*.*ch*, (PDB Code: 1EYX), shows that the Cα-Cα distance between Cys^50^ and Cys^61^ is 15.3Å, because both cysteines are part of a helical region. [8]. In γ^33^ subunit, this DL-PUB^62/73^, is also located in a helical region according to our results on prediction of secondary structure, and also according to the model proposed by Zhang *et al*. (2017)[12] from cryo-electron microscopy data. Fig 7 shows the structure of the inter cysteines region of the β subunit of R-PE from *G*.*ch* with β-DL-PUB^50/61^; the sequence for γ^33^-DL-PUB^62/73^ is also shown for comparison. Cys^73^, one of the linkages of PUB, is conserved in all sequences analyzed (Fig 4), however, it has not been considered as chromophorylation site previously [10, 12, 21]. Cys^62^ is present only on γ^33^ subunit from *G*. *ch*. This work shows clearly that both cysteines are involved in the binding of DL-PUB. These double linked bilins have been reported for phycobiliproteins, but never before for γ-linkers.

## Conclusion

The γ33 subunit of R-PE is a protein of 279 residues organized in 2 internal repeats that suggest its arrangement as domains. The molecule presents a chromophorylation ratio PEB/PUB = 1 based on the spectroscopic characterization. The chromophorylated residues detected by mass spectrometry are Cys95 (SL-PUB), Cys172 (SL_PEB) and Cys62/Cys 73 for a DL-PUB. According to cysteines conservation, evidences of chromophorylation and spectroscopy, and to complete the ratio PEB/PUB, the remaining position should be Cys259 (SL-PEB). The spectroscopy also indicates that there is not energy transfer between the different chromophores, supporting their contribution to the energy transfer with R-PE along the antenna. Moreover, we are reporting the first evidence of a DL-PUB in a linker protein in the phycobilisome system.

## Supporting information

S1 Table Mass list MS (m/z) experimentally obtained after treatment of γ^33^ subunit with trypsin and cyanogen bromide (CNBr). Mass list MS (m/z) experimentally obtained after treatment of γ^33^ subunit with trypsin and cyanogen bromide (CNBr) and used in the search by MS-Bridge in http://prospector.ucsf.edu/prospector/cgi-bin/msform.cgi?form=msbridgestandard.
(DOCX)
