# Correction: The γ^33^ subunit of R-phycoerythrin from *Gracilaria chilensis* has a typical double linked phycourobilin similar to β subunit

**DOI:** 10.1371/journal.pone.0200867

**Published:** 2018-07-13

**Authors:** Aleikar Vásquez-Suárez, Francisco Lobos-González, Andrew Cronshaw, José Sepúlveda-Ugarte, Maximiliano Figueroa, Jorge Dagnino-Leone, Marta Bunster, José Martínez-Oyanedel

There is an error in the title. The correct title is: The γ^33^ subunit of R-phycoerythrin from *Gracilaria chilensis* has a typical double linked phycourobilin similar to β subunit. The correct citation is: Vásquez-Suárez A, Lobos-González F, Cronshaw A, Sepúlveda-Ugarte J, Figueroa M, Dagnino-Leone J, et al. (2018) The γ^33^ subunit of R-phycoerythrin from Gracilaria chilensis has a typical double linked phycourobilin similar to β subunit. PLoS ONE 13(4): e0195656. https://doi.org/10.1371/journal.pone.0195656.
