# Supplementary material for: The γ33 subunit of R-phycoerythrin from Gracilaria chilensis has a typical double linked phycourobilin similar to γ subunit
Source: PLoS One. 2018 Apr 10;13(4):e0195656. doi: 10.1371/journal.pone.0195656 (PMC5892909; doi:10.1371/journal.pone.0195656)
Supplement: S1 Table — Mass list MS (m/z) experimentally obtained after treatment of γ33 subunit with trypsin and cyanogen bromide (CNBr) and used in the search by MS-Bridge in http://prospector.ucsf.edu/prospector/cgi-bin/msform.cgi?form=msbridgestandard. (DOCX) [file pone.0195656.s001.docx]

**Supporting material**.

TABLE: Mass list MS (m/z) experimentally obtained after treatment of γ^33^ subunit with trypsin and cyanogen bromide (CNBr) and used in the search by MS-Bridge in http://prospector.ucsf.edu/prospector/cgi-bin/msform.cgi?form=msbridgestandard.

| m/z |  |
| --- | --- |
| 719,2 |  |
| 726,1 |  |
| 768,3 |  |
| 788,9 |  |
| 801,9 |  |
| 803,2 |  |
| 840,8 |  |
| 876,8 |  |
| 914,2 |  |
| 919,2 |  |
| 927,4 |  |
| 931,3 |  |
| 971,3 |  |
| 1065,8 |  |
| 1069,4 |  |
| 1073,4 |  |
| 1075,1 |  |
| 1088,8 |  |
| 1165,5 |  |
| 1185,5 |  |
| 1249,5 |  |
| 1279,5 |  |
| 1319,5 |  |
| 1493,6 |  |
| 1537,6 |  |
| 1582,6 |  |
| 1585,7 |  |
| 1669,7 |  |
| 1685,8 |  |
| 1757,7 |  |
| 1845,7 |  |
| 2109,8 |  |
| 2197,9 |  |
| 2232,6 |  |
| 2285,9 |  |
| 2329,9 |  |
| 2373,9 |  |
| 2401,7 |  |
| 2464 |  |
| 2484,6 |  |
| 2551 |  |
| 2594 |  |
| 2703,8 |  |
| 2727,1 |  |
| 2816,1 |  |
| 2859,1 |  |
| 2904,2 |  |
| 2955,2 |  |
| 3036,2 |  |
| 3123,3 |  |
| 3212,3 |  |
| 3297,3 |  |
| 3298,4 |  |
| 3388,4 |  |
| 3444 |  |
| 3475,5 |  |
